# An adaptive slicing algorithm based on model contour information

**DOI:** 10.1371/journal.pone.0344508

**Published:** 2026-04-08

**Authors:** Xingguo Han, Xuan Liu, Kunteng Lu, Lixiu Cui

**Affiliations:** 1 Guangxi Key Laboratory of Special Engineering Equipment and Control, Guilin University of Aerospace Technology, Guilin, Guangxi, China; 2 School of Mechanical and Electrical Engineering, Guilin University of Electronic Technology, Guilin, Guangxi, China; 3 Key Laboratory of Special Engineering Equipment Design and Intelligent Driving Technology, Education Department of Guangxi Zhuang Autonomous Region, Guilin University of Aerospace Technology, Guilin, Guangxi, China; University of Perugia: Universita degli Studi di Perugia, ITALY

## Abstract

The original slicing algorithm needs to traverse all triangular facets when performing triangular facet screening, and the calculation amount is large. When performing model feature recognition, the feature standard is less, resulting in inaccurate feature recognition and the phenomenon that the position of the model feature moves. To solve these problems, an adaptive hierarchical algorithm based on model contour information is proposed. This algorithm is applicable to fused deposition additive manufacturing. By analyzing the geometric characteristics of the STL (Standard Triangulated Language) model, the model is divided into non-significant feature part, general feature part and complex feature part by using the change of contour intersection point number. For the model with non-significant feature part, the angle between the normal vector of the triangular patch and the stratification direction is used as the basis of slicing. For the model of the general feature part, the angle between the normal vectors of the adjacent triangular patches in this part is used as the basis of slicing algorithm. For the model of complex feature part, the index based on Taubin vertex normal vector is used as the basis of slicing algorithm. The equal thickness slicing algorithm, the adaptive slicing algorithm based on the normal vector of triangular facets and the algorithm proposed in this paper are used to slice the ship model, vase model and dice model respectively. The models are printed by the six-degree-of-freedom (six-DOF) additive manufacturing (AM) device and tested by a roughness meter. The experimental results show that the proposed method can better balance the printing accuracy and printing efficiency.

## Introduction

Additive manufacturing, also known as 3D printing, is a manufacturing method based on the principle of discrete-stacking, which constructs a three-dimensional (3D) entity by layer-by-layer superposition of materials. This technology has the characteristics of short manufacturing cycle, high material utilization rate and strong design flexibility [1]. Therefore, it has a wide application prospect and has applications in industrial production [2,3], biomedicine [4], aerospace [5] and other aspects.

**Fig 1:**
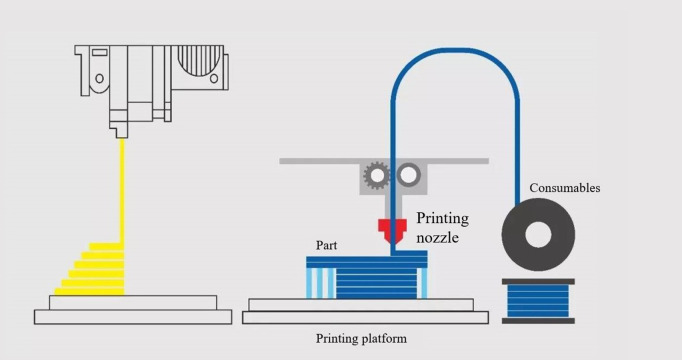
The principle of AM slicing algorithm.

In the process of AM, the slicing algorithm is one of the core links. It is responsible for converting the 3D model into a machine-recognizable instruction file. These instruction files control the printing nozzle to stack the material layer by layer, and finally form the part. The principle of the slicing algorithm is shown in Fig 1. The efficiency and accuracy of the slicing algorithm directly affect the fabrication speed, material consumption, and the accuracy and quality of the part.

The slicing algorithm of AM can be divided into plane slicing algorithm and surface slicing algorithm. Domestic and foreign scholars have carried out relevant research on these methods.

In the terms of plane slicing algorithm based on STL model, the adaptive slicing algorithm proposed by Yi et al. [6] and Li et al. [7] are based on the change of model contour number as the feature judgment basis. The proposed algorithm slows down the staircase effect and the influence of model feature loss and offset on printing quality, but the algorithm has a large amount of calculation, and the slicing effect is poor for a single contour model with complex features. The adaptive stratification algorithm proposed by Zhu et al. [8] is similar to the reference [6]. Compared with the reference [6], the proposed method considers the situation more comprehensively and the stratification effect is better. The algorithm proposed by Pan and Chen et al. [9,10] reconstructs the topological information according to the relationship between the triangular patch point table and the face table of the STL model, calculates the intersection of the triangular patch and the tangent plane, and adjusts the layer thickness according to the area change ratio of the adjacent tangent plane. This method is only applicable to the case where the adjacent tangent plane changes, and there is also a problem of cumbersome calculation. The algorithm proposed by Li et al. [11] determines the layer thickness according to the contour curvature of the STL model. When the contour curvature of the model is large, a smaller layer thickness is selected, and when the contour curvature of the model is small, a larger layer thickness is selected, but there will still be a model feature offset problem. Han et al. [12] proposed an adaptive slicing algorithm based on the feature information of STL model. This method can preserve the feature details of the model well, but does not consider the influence of the staircase effect on the model.

In terms of curve slicing algorithm, Erkan Gunpinar [13] proposed a curvature and feature-aware additive manufacturing printing path planning algorithm based on hexahedral meshes. The generated printing path can better fit the geometric features of the model, reduce the stair-stepping effect, and improve the mechanical properties of the printed parts. Serhat Cam et al. [14] proposed a curvature-aware printing path generation method based on fluid flow, which can effectively reduce the cross-contact between printing paths and improve the printing accuracy of the model. Liu et al. [15] proposed a multi-axis 3D printing slicing method based on neural network, which can generate a surface layer that meets the requirements of no support and strength enhancement. Yan et al. [16] proposed a streamlined printing path planning method based on the principal stress direction. The principal stress direction field was extracted by finite element analysis, and the streamline was generated based on this as the printing path, which significantly improved the mechanical properties of the printed parts.

In this paper, the plane layering algorithm of additive manufacturing will be studied. According to the above analysis of the existing plane layering algorithm, the current plane layering algorithm has the following two problems: First, the triangular patch retrieval has a large amount of calculation. Secondly, in feature recognition, the feature judgment standard used cannot accurately identify features. For example, the change of the number of cross-section contours is used as the feature judgment standard. The practicability of this feature judgment standard on a single contour model is not prominent.

**Fig 2:**
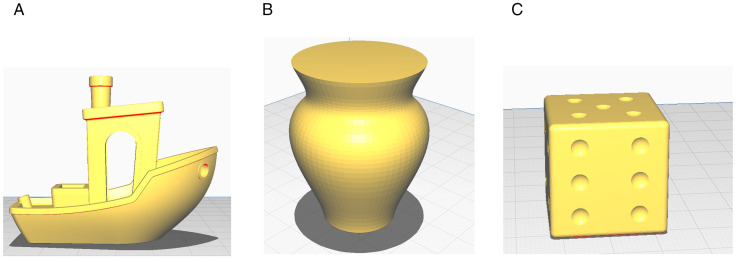
Models. A: Ship. B: Vase. C: Dice.

Aiming at these problems, an adaptive slicing algorithm based on model contour information is proposed. Firstly, the face table and the normal vector table are established to facilitate the screening of qualified triangular facets. Secondly, according to the contour feature of the model, that is, the change of the number of intersection points, the model is divided into non-significant feature part, general feature part and complex feature part by setting the threshold of the change of the number of intersection points. For the model with non-significant feature part, the angle between the normal vector of the triangular patch and the stratification direction is used as the basis of slicing algorithm. For the model of the general feature part, the angle between the normal vectors of the adjacent triangular patches in this part is used as the basis of slicing algorithm. For the model of complex feature part, the index based on Taubin vertex normal vector is used as the basis of slicing algorithm. Finally, the layered slicing of the model is completed and the printing experiment is completed. In this paper, the vase model, the dice model and the ship model are sliced by using the equal thickness slicing algorithm (ETSA), the adaptive slicing algorithm based on the normal vector of triangular patches (ASANV) and the proposed algorithm. The models are modeled by using the 3D modeling software SOLIDWORKS 2018. The models are shown in Fig 2. The models are printed by the six-DOF AM device and tested by the surface roughness meter. Experiments show that the proposed algorithm is simple and effective and practical in improving the quality of model fabrication.

## STL model information reading and storage

**Fig 3:**
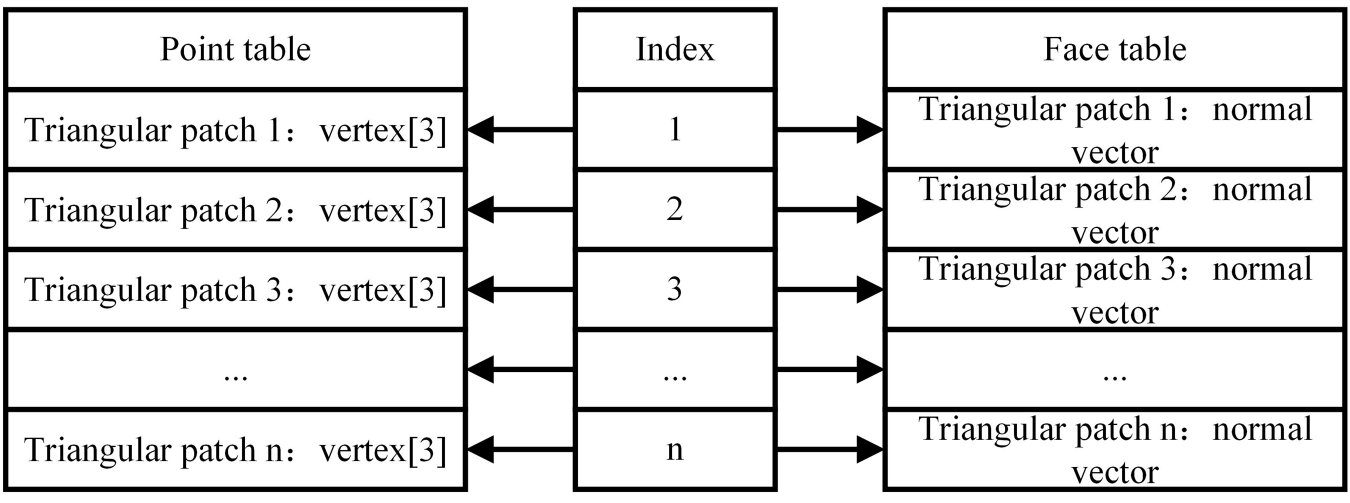
Storage structure.

Before the adaptive slicing process, the data of the STL model needs to be extracted and used as the basis for subsequent calculations. Because the STL file processing function built-in MATLAB cannot accurately obtain the vertex coordinates of the triangular patches, a custom function ‘stlread1’ was written to achieve this function. The function analyzes the triangular patches in the STL file one by one, and uses a hash table to eliminate duplicate data, and then constructs a point table and a face table to store the corresponding information. The storage structure is shown in Fig 3. The point table records the vertices of the triangular patches in detail, and the face table stores the normal vectors of each triangular patch. The reading and storage method of this model not only facilitates the reconstruction of the topology between vertices and faces, but also helps to reduce data redundancy and improve computational efficiency [17].

## Analysis of adaptive slicing algorithm

The direct factor affecting the fabrication quality of the model is the staircase effect. Staircase effect is a common surface defect in AM, especially in fused deposition modeling (FDM). This effect is caused by layer-by-layer stacking during the printing process. The edge of each layer of the model has obvious staircases in the vertical direction, which affects the surface quality of the model.

### Adaptive slicing algorithm based on the normal vector of triangular patches

**Fig 4:**
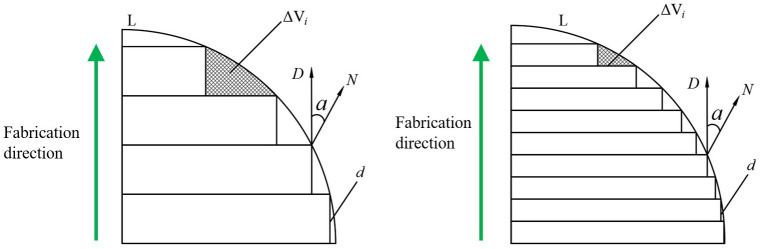
Staircase effect.

From Fig 4, it can be seen that the fabrication direction of STL model has a certain relationship with the angle α of the normal vector of triangular patches at this point and the printing geometric error $\Delta V_{i}$ of the layer *i*: the larger the α, the smaller the $\Delta V_{i}$. α is determined by the STL model’s own contour, and cannot be changed for a triangular patch. Therefore, the relationship between α and layer thickness *d* can be established, and the geometric error $\Delta V_{i}$ can be reduced by changing the layer thickness reasonably, so as to improve the printing accuracy.

**Fig 5:**
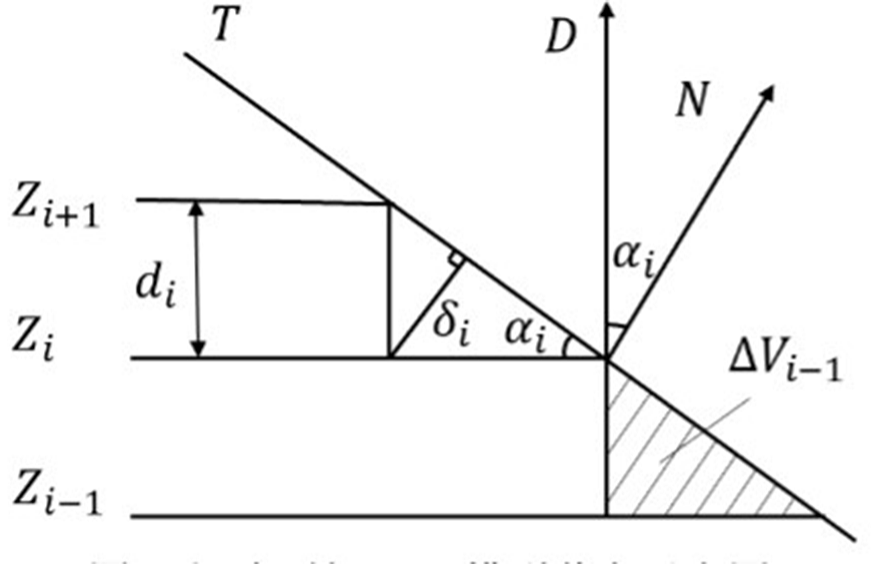
Intersection of sliced surface and STL model.

When the printing layer thickness changes, the corresponding geometric error $\Delta V_{i}$ will also change. Because the geometric error $\Delta V_{i}$ in reference [18] is complex and time-consuming, the staircase height $\delta_{i}$ in reference [19] is introduced as the evaluation index of the geometric error of the model.  Fig 5 is a schematic diagram of the intersection of the adjacent tangent planes *Z*_*i*_ and *Z*_*i*+1_ with the STL model surface *T*. Where *T* is the surface of the model, *D* is the fabrication direction of the model, *N* is the normal vector of the triangular patches of the STL model at this point, α is the angle between the printing fabrication direction of the layer *i* model and the normal vector of the triangular patches of the STL model at this point, $\delta_{i}$ is the staircase height of the model, *d*_*i*_ is the thickness of the layer *i*.

From Fig 5, the angle $\alpha_{i}$ between the fabrication direction *D* of the STL model of the layer *i* and the normal vector of the triangular patches at this point can be obtained.

When $\alpha_{i}>90^{\circ }$, then $\alpha_{i}=180-\alpha_{i}$, so the value range of $\alpha_{i}$ is [0,90]. When $\alpha_{i}$ decreases, the staircase height $\delta_{i}$ increases, and the model fabrication accuracy decreases. The staircase effect can be slowed down by reducing the layer thickness, that is, when $\alpha_{i}$ decreases, the layer thickness *d*_*i*_ also decreases. When $\alpha_{i}$ increases, the staircase height $\delta_{i}$ decreases, and the fabrication quality of the model increases. That is, when $\alpha_{i}$ increases, the layer thickness *d*_*i*_ also increases. According to this relationship, it can be expressed by the following formula. In the formula, *y* represents the relationship between the layer thickness and the angle. The angle refers to the angle between the fabrication direction and the normal vector of the triangular patch.


$$y=1-cos\alpha_{i}$$ (1)

**Fig 6:**
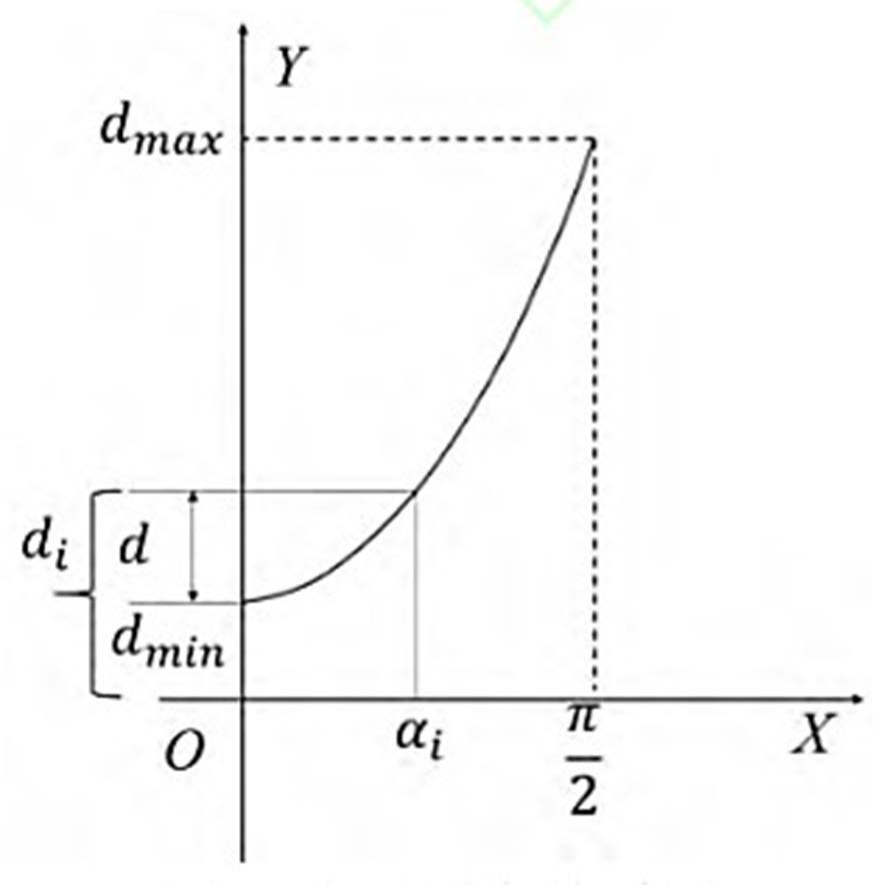
Relationship between angle and layer thickness.

According to the requirements of equipment and processing technology, it is necessary to set the layer thickness range. According to the relationship between and, the corresponding relationship between the angle between the fabrication direction of the layer i and the normal vector of the triangular patches at this point and the thickness of the layer i can be obtained. The relationship is shown in Fig 6. The calculation formula of the layer thickness of ASANV is shown as follows.


$$d_{i}=d_{\min}+(d_{\max}-d_{\min})(1-\cos\alpha_{i})$$ (2)

### An adaptive slicing algorithm based on model contour information

#### Section contour calculation.

**Fig 7:**
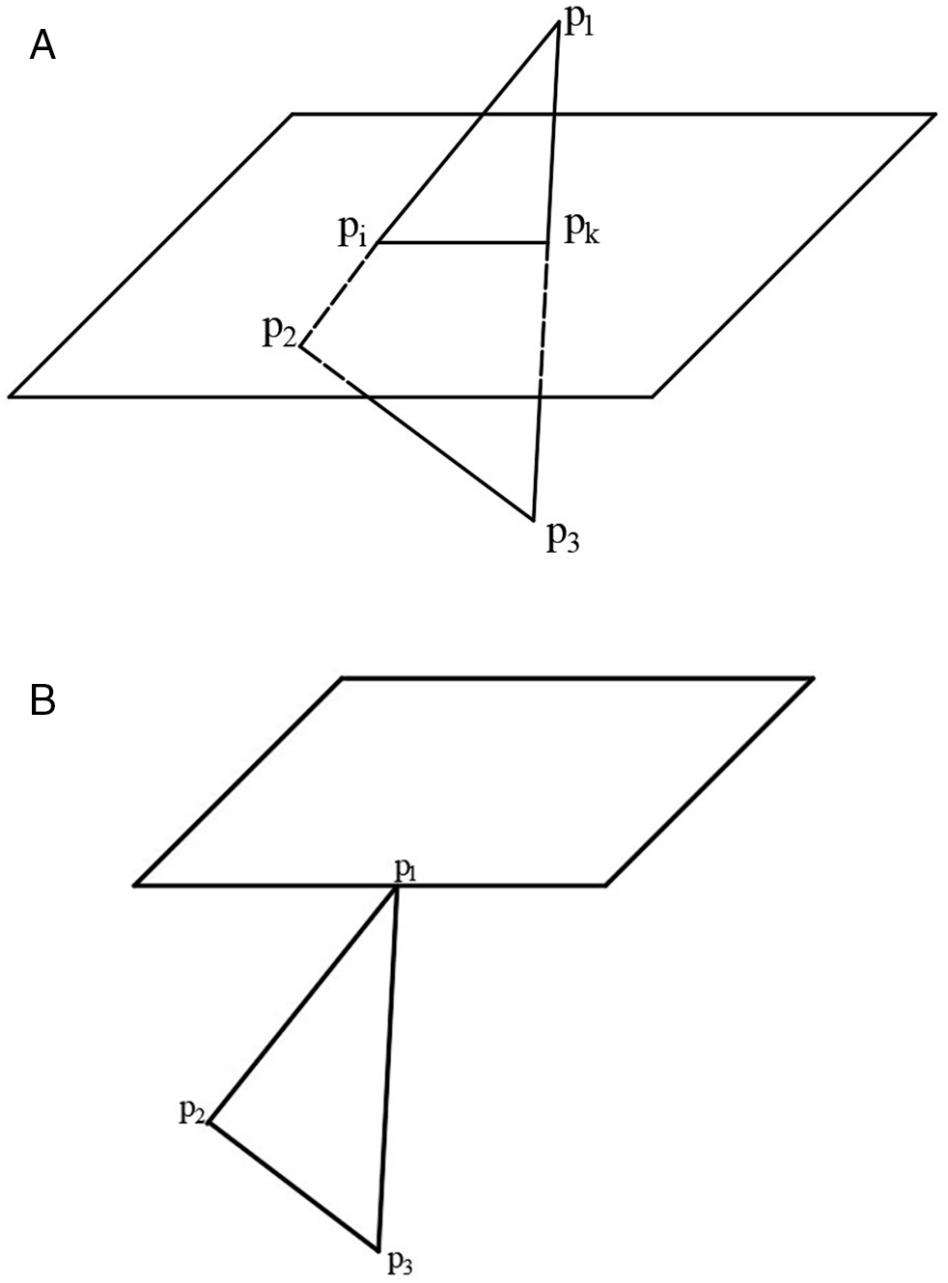
Triangular patches intersect with the slicing plane. A: General case. B: Special case.

In order to ensure the fabrication accuracy of the model, the slicing thickness of the STL model is small, and a triangular patch often needs to intersect with multiple adjacent slicing planes. For the intersection calculation of triangular facets and slicing planes, as shown in Fig 7(A), let the slicing plane intersect with the triangular patches, and the intersection points are *p*_*i*_ and *p*_*k*_, respectively. The three vertices of the triangular patches are $p_{1}(x_{1},y_{1},z_{1})$, $p_{2}(x_{2},y_{2},z_{2})$ and $p_{3}(x_{3},y_{3},z_{3})$. The equation of the straight line $p_{1}p_{2}$ is as follows.


$$\frac{x_{i}-x_{1}}{x_{2}-x_{1}}=\frac{y_{i}-y_{1}}{y_{2}-y_{1}}=\frac{z_{i}-z_{1}}{z_{2}-z_{1}}$$ (3)

The coordinates of points are as follows.


$$\left\{ \begin{array}{c} x_{i}=\frac{x_{2}-x_{1}}{z_{2}-z_{1}}(z_{i}-z_{1})+x_{1}\\ y_{i}=\frac{y_{2}-y_{1}}{z_{2}-z_{1}}(z_{i}-z_{1})+y_{1}\\ z_{i}=z_{i} \end{array}\right.$$ (4)

Similarly, the coordinates of the intersection point *p*_*k*_ can be obtained by calculation. Through the above formula, the coordinate values of each intersection point when the triangular patch intersects with each slicing plane can be calculated. In order to save storage space and improve the efficiency of subsequent operations, for the case where the vertices of the triangular patches are intersections, as shown in Fig 7(B), only one of *p*_*i*_ and *p*_*k*_ should be retained.

#### Judgment standard of features.

Although the adaptive slicing algorithm based on the normal vector of triangular patches can effectively alleviate the staircase effect and improve the accuracy of the model, due to the incomplete criteria of feature judgment, there may be some phenomena such as the offset of model features.

**Fig 8:**
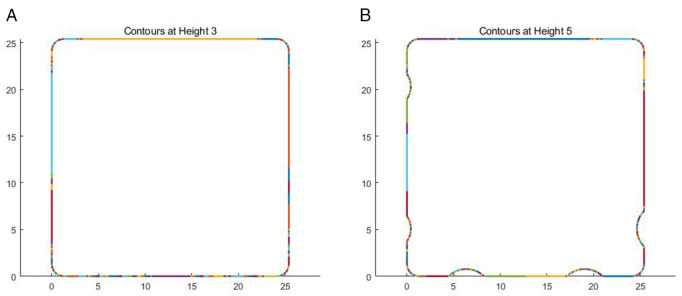
Cross-sectional profile of the dice. A: Contours at height 3. B: Contours at height 5.

The change of the surface feature of the model inevitably leads to the change of the number of cross-section contour points, and the more complex the feature is, the more triangular patches are needed to describe the feature, and the more contour points are needed. The number of contour intersections in different contours is shown in Fig 8. The line segments of different colors in the figure represent the line segments connected by the intersections of different triangular patches under the current contour. As shown in Fig 8(A), there are 454 contour points in the cross-section contour of the dice at a layering height of 3 mm.  Fig 8B shows the cross-section contour of the dice at a layering height of 3 mm, with a total of 734 contour points. Therefore, in the proposed algorithm, the change value of the number of edges between the imaginary surface and the intersecting triangular patches is greater than a certain threshold, which is used as the basis for model feature judgment. In order to ensure the accuracy of feature judgment, the number of intersection points of the current layer is compared with the number of intersection points of adjacent slicing planes. If it is an arithmetic sequence, it is regarded as a non-significant feature, as shown in Eq 5.


$$\left\|P_{i-1}-P_{i-2}\right\|-\left\|P_{i-2}-P_{i-3}\right\|=0$$ (5)

In the formula, $P_{i-1}$, $P_{i-2}$ and $P_{i-3}$ are the number of contour intersection points of the three adjacent layers, respectively, *i* > 3.

β is set as the contour focus change value. If the change value of the number of intersection points between the i-1 layer and the contour of the i layer is a, and the change value of the number of intersection points between the i-1 layer and the contour of the i-2 layer is b, then β is the difference between a and b. β can be obtained by Eq 6.


$$\left|\left|P_{i}-P_{i-1}\right|-\left|P_{i-1}-P_{i-2}\right|\right|=\beta$$ (6)

The change threshold of the contour intersection point is set to *B*. By setting the parameter, the recognition accuracy of the model can be effectively improved, so the parameter effectively reflects the degree of change of the model features. The model features can be divided into general features and complex features, and the criterion of the two is the range of the difference. When β∈(0,B], it is a general feature. When β∈[B,∞], it is a complex feature.

#### Determination of adaptive layer thickness.

For the non-significant feature part, an adaptive slicing algorithm based on triangular patch normal vector is used to hierarchically process this part of the model. The principle of the algorithm is described in Section ‘Adaptive Slicing Algorithm Based on The Normal Vector of Triangular Patches’. The calculation formula of layer thickness is as follows.


$$h_{1}^{\prime }=d_{\min}+(d_{\max}-d_{\min})(1-\cos\alpha_{i})$$ (7)

Where $d_{\min}$ is the minimum layer thickness and $d_{\max}$ is the maximum layer thickness.

**Fig 9:**
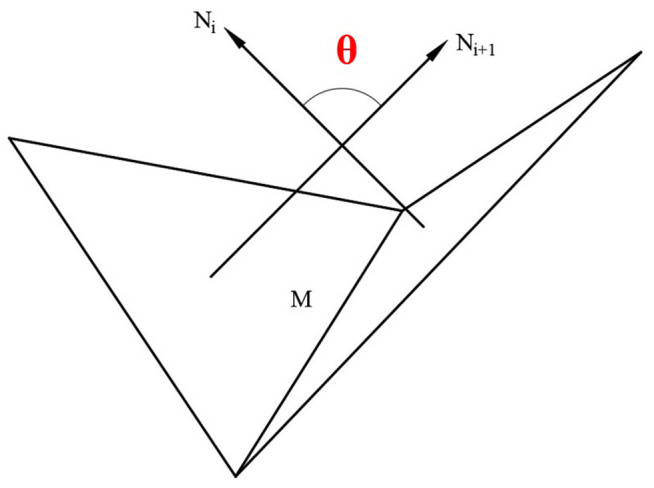
The angle diagram of adjacent triangular facets.

For the general feature part, the angle between the normal vectors of adjacent triangular patches is used as the basis of slicing algorithm.  Fig 9 is a schematic diagram of the angle between two triangular patches containing a common edge M, where the normal vectors of the two triangular patches are *N*_*i*_ and *N*_*i*+1_, respectively, and the angle between the two normal vectors is θ.

The relationship between θ and layer thickness: when θ increases, the characteristics of the model are more significant, that is, the ideal layer thickness is smaller. When θ decreases, the characteristics of the model are not significant, that is, the ideal layer thickness can be appropriately increased, and the calculation formula of layer thickness is as follows.


$$h_{2}^{\prime }=d_{\max}-(d_{\max}-d_{\min})\left(\frac{1-\cos\theta }{2}\right)$$ (8)

**Fig 10:**
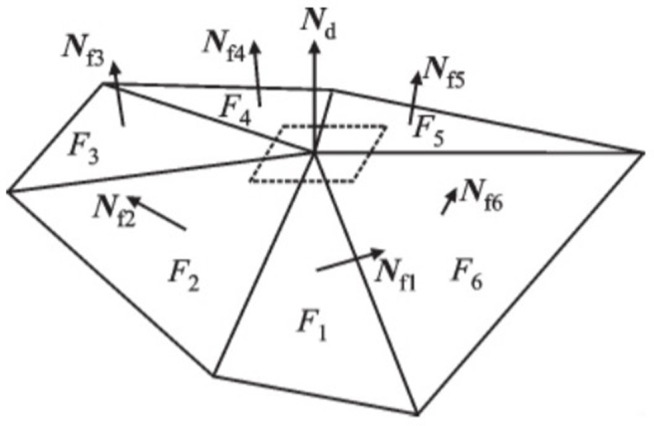
Vertex normal vector.

For the complex feature part, an index based on the Taubin vertex normal vector that can reflect the complexity of the current part is used for calculation [20]. The complex features in the STL model are composed of multiple triangular patches. The vertex normal vector at the complex feature can reflect the direction of the vertex. As shown in Fig 10, *N*_*d*_ is the vertex normal vector of the complex feature part, and *F*_*i*_ is all triangular patches adjacent to the feature.

The normal vector of Taubin vertex is the weighted average of the normal vectors of adjacent triangular patches with area as the weight. The estimation formula is shown in Eq 10.


$${𝐍}_{d}^{\prime }=\frac{\sum_{i=1}^{n}S_{i}{𝐍}_{fi}}{\sum_{i=1}^{n}S_{i}};{𝐍}_{d}={𝐍}_{d}^{\prime }/|{𝐍}_{d}^{\prime }|$$ (9)

In the formula, *S*_*i*_ is the area of triangular patches, *N*_*fi*_ is the normal vector of triangular patches, $N_{d}^{\prime }$ is the intermediate variable.

After obtaining the vertex normal vector, the average direction feature at the complex feature is obtained. The complexity of the complex feature is related to the angle between the normal vector of each triangular patch and the normal vector of the vertex. The larger the angle is, the more complex the complex feature is. Considering the effect of all triangular patches at the complex feature, r can be used as an index to judge the complexity of the feature. The calculation formula is as follows.


$$r=\frac{\sum_{i=1}^{n}S_{i}|{𝐍}_{d}•{𝐍}_{fi}|}{\sum_{i=1}^{n}S_{i}}$$ (10)

*r* can be used to truly reflect the complexity of the complex features of the model. The smaller the value of *r*, the more complex the model is here. The calculation formula of layer thickness is as follows.


$$h_{3}^{\prime }=d_{\min}+(d_{\max}-d_{\min})r$$ (11)

In order to ensure the controllability of the layering fineness, λ can be set according to the allowable range of the layer thickness of the printer to further improve the printing accuracy. The range can be obtained by the following formula.


$$\left\{ \begin{array}{c} \lambda_{\min}=\max\left(\frac{d_{\max}}{\text{upper\_limit}},\frac{d_{\min}}{\text{lower\_limit}}\right)\\ \lambda_{\max}=\max\left(\frac{\text{upper\_limit}}{d_{\max}},\frac{\text{lower\_limit}}{d_{\min}}\right) \end{array}\right.$$ (12)

The upper limit of the layer thickness of the additive manufacturing device is 0.2 mm, the lower limit is 0.1 mm, and the maximum printing layer thickness and the minimum printing layer thickness set at the layering are 0.2 mm and 0.1 mm, respectively, then λ = 1.

The modified three types of adaptive stratification formulas are as follows.


$$h_{1}=\lambda (d_{\min}+(d_{\max}-d_{\min})(1-\cos\alpha_{i}))$$ (13)

$$h_{2}=\lambda (d_{\max}-(d_{\max}-d_{\min})(\frac{1-\cos\theta }{2}))$$ (14)

$$h_{3}=\lambda (d_{\min}+(d_{\max}-d_{\min})r)$$ (15)

When the layer height changes, in order to ensure the printing quality, based on the equal volume method, that is, the volume of the consumables sent to the consumables conveying mechanism is equal to the volume of the extruded part. To ensure that the motor speed of the consumables conveying mechanism is constant, the moving speed of the manipulator is changed to adapt to different layer heights, and the speed of the manipulator can be obtained by Eq 16.


$$V_{M}=\frac{V_{C}\cdot d_{c}^{2}}{h^{2}}$$ (16)

In the Eq 16, *V*_*M*_ is the moving speed of the manipulator, *V*_*C*_ is the motor speed of the consumables conveying mechanism, *d*_*c*_ is the diameter of the consumables, and *h* is the height of the current layer.

#### Algorithm procedure.

**Fig 11:**
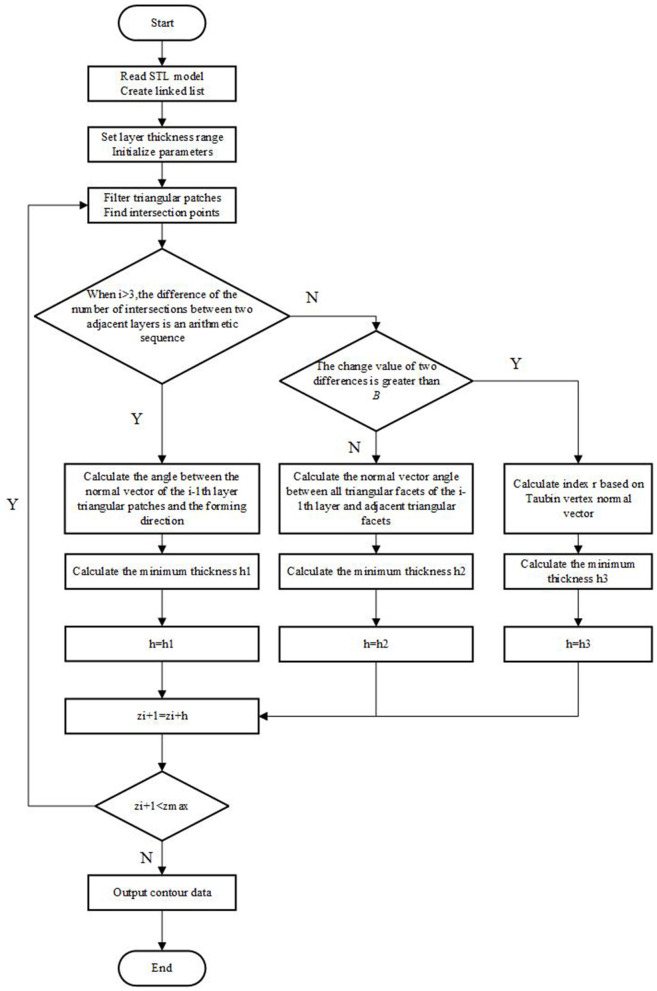
Algorithm flow chart.

In the process of AM, the initial layer usually has an attached support structure to ensure a stable connection between the model and the printing platform to reduce the warpage of the model. Therefore, the initial layer thickness is generally artificially set. The layer thickness of the second layer and the third layer is 0.1. The adaptive layering starts from the fourth layer of the model. The adaptive layering algorithm flow in this paper is shown in Fig 11.

Step1: Read the STL model information and establish a linked list. Input the printing layer thickness range *d*_*max*_,*d*_*min*_. After reading the STL model, the most size value of the model can be obtained. The initial layer height is set.

Step2: Traverse the triangular patches to find all the triangular patches that intersect with the layer *z*_*i*_. Through the judgment of features, it is judged whether there is a feature change in the layer *z*_*i*_. If there is no significant feature, the adaptive layer thickness *h*_1_ is calculated by Eq 13; if there are general characteristics, the adaptive layer thickness *h*_2_ is calculated by Eq 14; if there are complex features, the adaptive hair layer thickness *h*_3_ is calculated by Eq 15.

Step3: When *i* > 3, it is judged whether the difference between the *i*−1th layer and the first two layers is an arithmetic sequence. If it is an arithmetic sequence, then *h* = *h*_3_; if it is not an arithmetic progression, and the difference is less than B, then by *h* = *h*_3_; if it is not an arithmetic progression and the difference is greater than B, then *h* = *h*_3_.

Step4: Determine the current layer thickness *h*, then the layer height of the next STL model is $z_{i+1}=z_{i}+h$.

Step5: Determine whether the current layer height *z*_*i*_ exceeds the STL model height *z*_*max*_. If the height of the slicing plane exceeds the height of the model, the slicing ends, otherwise Step2 is returned to continue the loop to determine the height of the next layer. When $z_{i}>z_{max}$, a series of two-dimensional cross-section contour curves are finally output.

## Experiment and analysis

**Fig 12:**
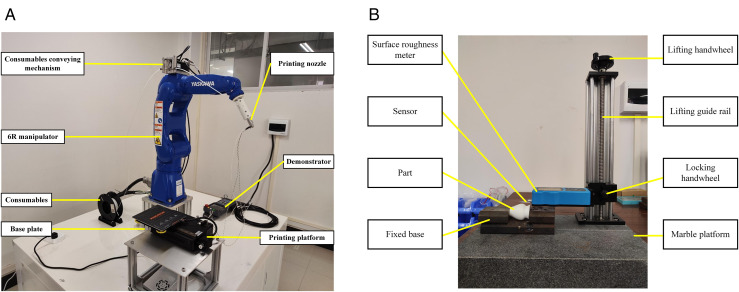
Experimental devices. A: Six-DOF AM device. B: Surface roughness meter.

According to the above algorithm principle, the experiment is carried out on Windows10 using MATLAB R2019b. The same STL model is processed and compared by using the ETSA, ASANV and the adaptive slicing algorithm proposed in this paper. The surface roughness is selected as the evaluation index of model accuracy, and the printing time is selected as the efficiency index of model printing. The thickness of 0.1 mm and 0.2 mm is selected for equal thickness stratification. The minimum layer thickness of adaptive layering is set to 0.1 mm, that is, *d*_*min*_ = 0.1 *mm*, and the maximum layer thickness is set to 0.2 mm, that is, *d*_*max*_ = 0.2 *mm*. The six-DOF AM device is used for printing. The surface roughness of the model was detected by a surface roughness meter. The experimental devices are shown in Fig 12. The relevant parameters of the experimental device are shown in Table 1. The relevant parameters of the surface roughness meter are shown in Table 2. The basic principle of the six-DOF additive manufacturing device is described in detail in References [21].

**Table 1:** Related parameters of six-DOF AM device.

Mechanical arm	Consumable materials	Diameter of consumables (mm)	Diameter of printing nozzle (mm)	Speed of printing sprinkler nozzle (mm/s)	Temperature of printing sprinkler (℃)	Temperature of printing platform (℃)
YASKAWA GP8	PLA	1.73	0.75	23.0	200	60

**Table 2:** Related parameters of surface roughness meter.

Key parameter	Size of measurement platform (mm)	Lifting height (mm)	Sampling length (mm)	Evaluation length (mm)	range (μ m)	Filtering mode
Value	400*250*70	350 ± 1	0.25,0.8,2.5	1.5,5	±20, ±80	GAUSS

**Fig 13:**
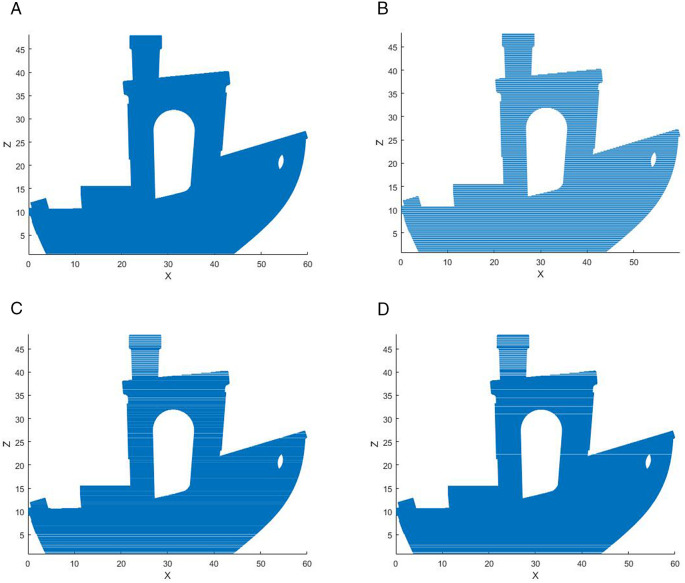
Slicing effect of the ship. A: ETSA(h = 0.1 mm). B: ETSA(h = 0.2 mm). C: ASANV. D: The proposed algorithm.

**Fig 14:**
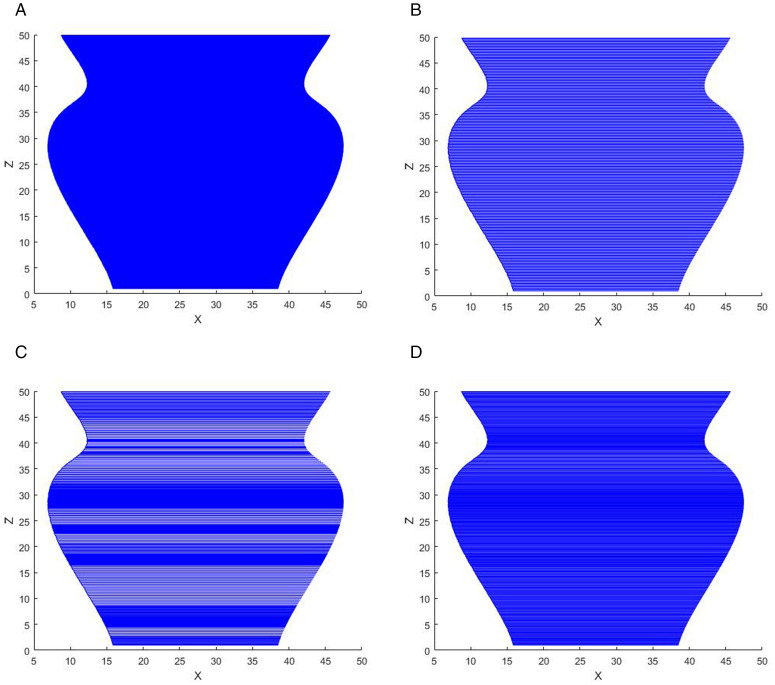
Slicing effect of the vase. A: ETSA(h = 0.1 mm). B: ETSA(h = 0.2 mm). C: ASANV. D: The proposed algorithm.

**Fig 15:**
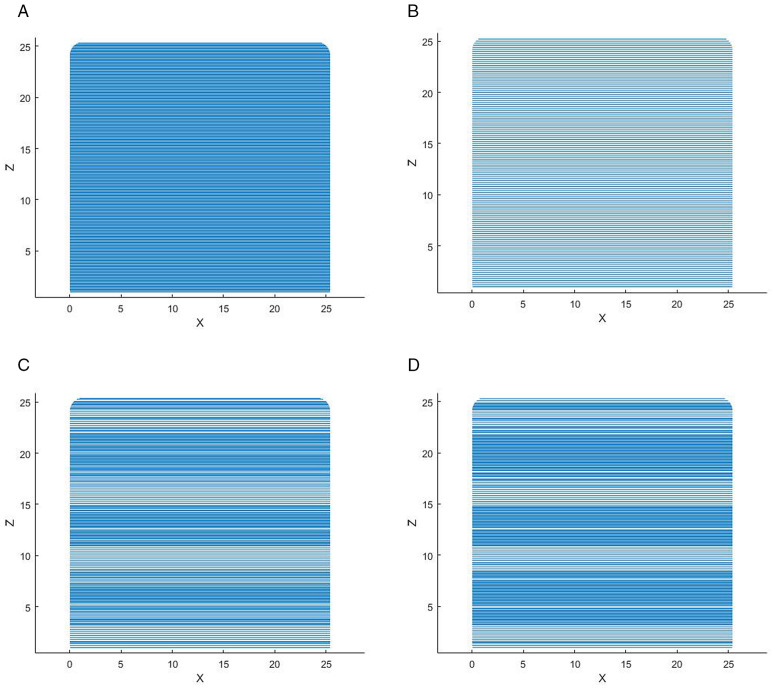
Slicing effect of the dice. A: ETSA(h = 0.1 mm). B: ETSA(h = 0.2 mm). C: ASANV. D: The proposed algorithm.

The initial layer thickness is set to 1 mm, λ = 1, B = 10, and the vase model is sliced using MATLAB 2019b. The slicing data of the model are shown in Tables 3–5, respectively, and the slicing effects are shown in  Figs 13–15, respectively.

**Table 3:** Slicing data of the ship.

Model	The number of triangular patches	The height of the model (mm)	Slicing algorithm	Layer thickness (mm)	Layer number
Ship	223710	48	ETSA	0.1	471
				0.2	236
			ASANV	0.1-0.2	405
			The proposed algorithm	0.1-0.2	423

**Table 4:** Slicing data of the vase.

Model	The number of triangular patches	The height of the model (mm)	Slicing algorithm	Layer thickness (mm)	Layer number
Vase	14688	50	ETSA	0.1	491
				0.2	246
			ASANV	0.1-0.2	263
			The proposed algorithm	0.1-0.2	297

**Table 5:** Slicing data of the dice.

Model	The number of triangular patches	The height of the model (mm)	Slicing algorithm	Layer thickness (mm)	Layer number
Dice	11274	25.4	ETSA	0.1	245
				0.2	123
			ASANV	0.1-0.2	187
			The proposed algorithm	0.1-0.2	195

It can be seen from Tables 3–5 and Figs 13–15 that the ETSA (h = 0.1 mm) has the highest slicing accuracy, and the ETSA (h = 0.2 mm) has the lowest slicing accuracy. Compared with the proposed method, in the key feature parts of the model, such as the bottleneck and the convex part of the bottle body, the layer thickness is small, and the large layer thickness is used as much as possible in other positions to improve the printing efficiency.

**Fig 16:**
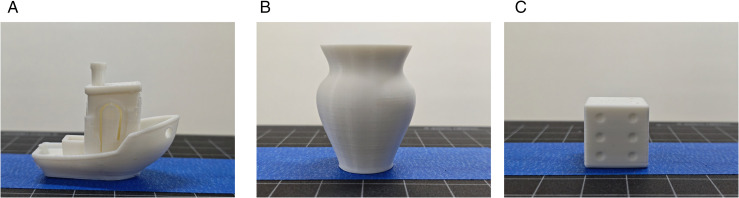
Parts. A: Ship. B: Vase. C: Dice.

**Fig 17:**
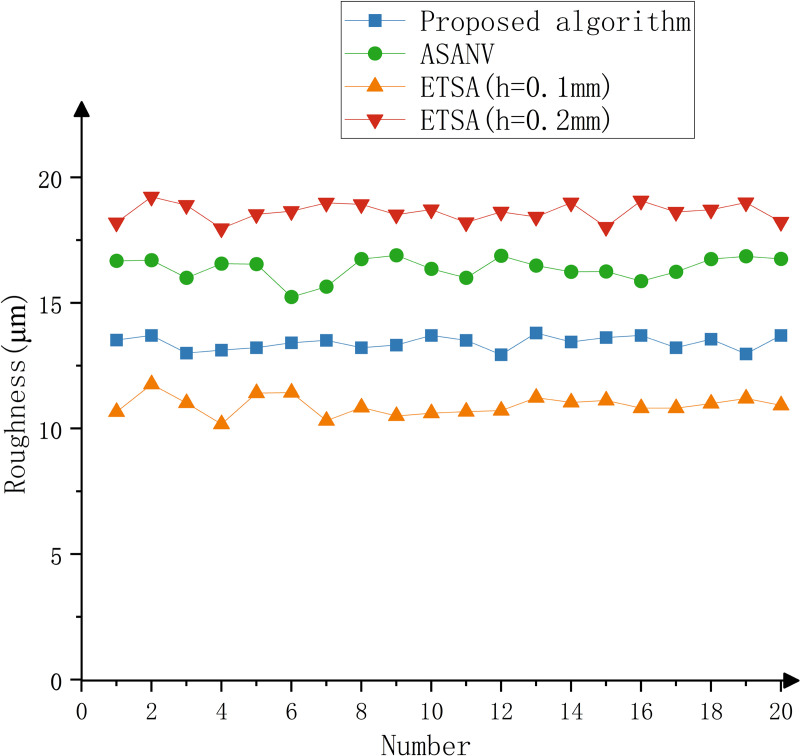
Experimental results of ship.

**Fig 18:**
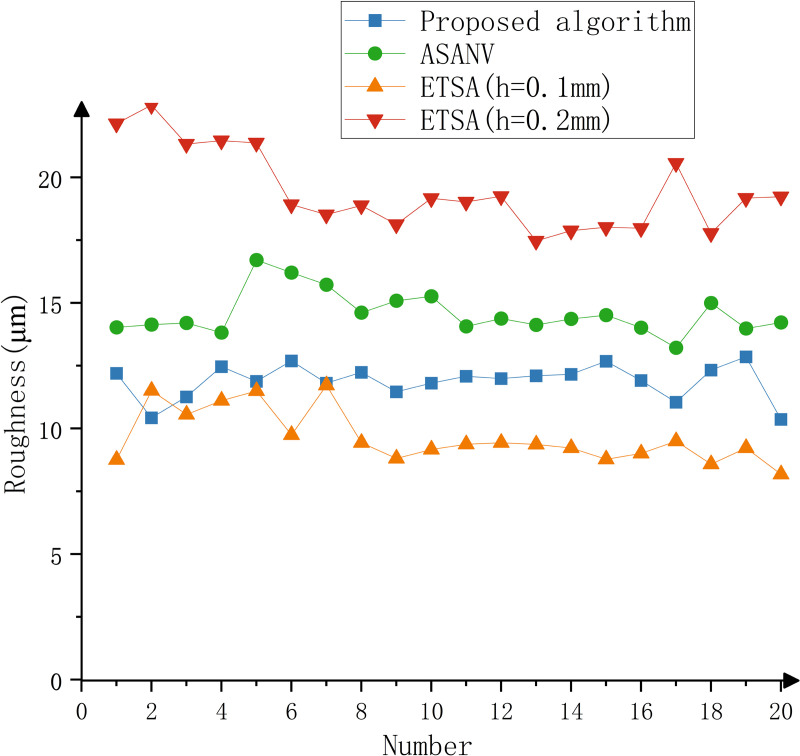
Experimental results of vase.

**Fig 19:**
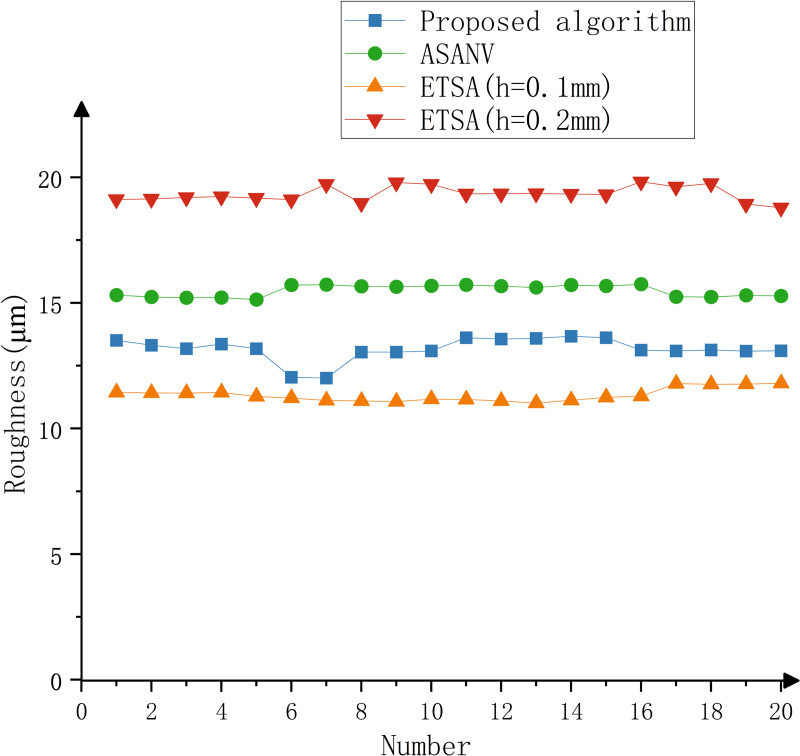
Experimental results of dice.

The model is printed by the six-DOF manipulator, and the printing time is recorded. The part is shown in Fig 16. The surface roughness meter is used to detect part, and the surface roughness of the key feature parts of the model is detected. A total of 20 points are selected. The experimental results are shown in Tables 6–8, Figs 17–19.

**Table 6:** Experimental results of ship.

Model	Slicing algorithm	Roughness (μm)	Time (min)
Ship	ESTA(h = 0.1 mm)	10.95505	193
	ESTA(h = 0.2 mm)	18.65715	96
	ASANV	16.38295	162
	The proposed algorithm	13.45015	171

**Table 7:** Experimental results of vase.

Model	Slicing algorithm	Roughness (μm)	Time (min)
Vase	ESTA(h = 0.1 mm)	9.69095	319
	ESTA(h = 0.2 mm)	19.4893	161
	ASANV	14.5821	175
	The proposed algorithm	11.92495	189

**Table 8:** Experimental results of dice.

Model	Slicing algorithm	Roughness (μm)	Time (min)
Dice	ESTA(h = 0.1 mm)	11.37845	118
	ESTA(h = 0.2 mm)	19.37355	60
	ASANV	15.48375	80
	The proposed algorithm	13.20525	92

It can be seen from the test results that when the ETSA is used to process the model, when the layer thickness is 0.1 mm, the surface roughness is the lowest, but the printing time is the longest. When the layer thickness is 0.2 mm, the surface roughness is the highest, and the corresponding printing time is the shortest. Compared with the ETSA (h = 0.1 mm), the surface roughness of the three models increased by 18.6%, 18.7% and 13.8% respectively, but the printing time was shortened by 22 min, 130 min and 26 min respectively. Compared with the ETSA (h = 0.2 mm), the surface roughness of the three models is reduced by 27.9%, 38.8% and 31.8% respectively, and the printing time is increased by 75 min, 28 min and 32 min respectively. Compared with the ASANV, the surface roughness of the three models is reduced by 17.9%, 18.2% and 14.7% respectively, and the printing time is increased by 9 min, 14 min and 12 min respectively. Compared with the other two algorithms, the algorithm proposed in this paper does not reduce the printing efficiency too much when the model fabrication quality is significantly improved. This efficiency loss is acceptable. The proposed method can better balance the printing accuracy and printing efficiency.

## Conclusion

In view of the problems existing in the current slicing algorithm, the algorithm in this paper fully considers the influencing factors of the model accuracy. It can use the proposed feature recognition method to mitigate the influence of the staircase effect on the model fabrication accuracy. The following three points are proved by experiments:

1. The method proposed in this paper is based on the model contour information, which is convenient for extraction and does not require complex curvature or closed contour calculation, so the algorithm complexity is small.
2. The change of the number of intersection points of the model contour is used as the basis for the feature judgment of the algorithm. Compared with the traditional slicing algorithm, it can better identify and retain the features, and alleviate the influence of the staircase effect on the model fabrication quality, so it has better practicability.
3. Through comparative experiments, the method proposed in this paper can effectively improve the fabrication accuracy of the model. Compared with the ETSA (h = 0.1 mm), the surface roughness of the three models increased by 18.6%, 18.7% and 13.8% respectively, but the printing time was shortened by 22 min, 130 min and 26 min respectively. Compared with the ETSA (h = 0.2 mm), the surface roughness of the three models is reduced by 27.9%, 38.8% and 31.8% respectively, and the printing time is increased by 75 min, 28 min and 32 min respectively. Compared with the ASANV, the surface roughness of the three models is reduced by 17.9%, 18.2% and 14.7% respectively, and the printing time is increased by 9 min, 14 min and 12 min respectively. It can better balance the printing accuracy and printing efficiency.

The limitations of this study mainly include the following two points:

1. The algorithm is based on STL model, and the application effect on other 3D models has not been proved. The algorithm will be further improved in the future to enhance the versatility of the algorithm.
2. For complex surface parts, surface layering is undoubtedly a better processing method. Subsequently, the surface layering will be studied, and combined with the six-DOF AM device, efficient supportless printing will be realized.

## Supporting information

S1 Data The minimal dataset. (XLSX)
